# Mapping Functional Associations in the Entire Genome of *Drosophila melanogaster* Using Fusion Analysis

**DOI:** 10.1002/cfg.287

**Published:** 2003-06

**Authors:** Ioannis Iliopoulos, Anton J. Enright, Patrick Poullet, Christos A. Ouzounis

**Affiliations:** 1 INA-EKETA Thessaloniki GR-57001 Greece; 2 Computational Biology Center Memorial Sloan–Kettering Cancer Center New York NY 10021 USA; 3 Institut Curie Paris F-75248 France; 4 Computational Genomics Group The European Bioinformatics Institute EMBL Cambridge Outstation Cambridge CB10 1SD UK

## Abstract

We have previously shown that the detection of gene fusion events can contribute towards the elucidation of functional associations of proteins within entire genomes. Here we have analysed the entire genome of *Drosophila melanogaster* using fusion analysis and two additional constraints that improve the reliability of the predictions, viz. low sequence similarity and low degree of paralogy of the component proteins involved in a fusion event. Imposing these constraints, the total number of unique component pairs is reduced from 18 654 to a mere 220 cases, which are expected to represent some of the most reliably detected functionally associated proteins. Using additional information from sequence databases, we have been able to detect pairs of functionally associated proteins with important functions in cellular and developmental pathways, such as spermatogenesis and programmed cell death.


**Comparative and Functional Genomics**
*Comp Funct Genom* 2003; **4**: 337–341.
Published online in Wiley InterScience (www.interscience.wiley.com). **DOI:** 10.1002/cfg.287

**Short Communication**

### Mapping functional associations in the entire genome of *Drosophila melanogaster* using fusion analysis

Ioannis Iliopoulos[1], Anton J. Enright[2], Patrick Poullet[3] and Christos A. Ouzounis[4]*
[1]*INA-EKETA, GR-57001 Thessaloniki, Greece*
[2]*Computational Biology Center, Memorial Sloan–Kettering Cancer Center, New York, NY 10021, USA*
[3]*Institut Curie, F-75248 Paris, France*
[4]*Computational Genomics Group, The European Bioinformatics Institute, EMBL Cambridge Outstation, Cambridge CB10 1SD, UK*

**Correspondence to:
Christos A. Ouzounis,
Computational Genomics Group,
The European Bioinformatics
Institute, EMBL Cambridge
Outstation, Cambridge CB10
1SD, UK.
E-mail: ouzounis@ebi.ac.uk*

Received: 10 July 2002
Revised: 17 February 2003
Accepted: 17 February 2003

### Abstract

**We have previously shown that the detection of gene fusion events can contribute towards the elucidation of functional associations of proteins within entire genomes. Here we have analysed the entire genome of *Drosophila melanogaster* using fusion analysis and two additional constraints that improve the reliability of the predictions, viz. low sequence similarity and low degree of paralogy of the component proteins involved in a fusion event. Imposing these constraints, the total number of unique component pairs is reduced from 18 654 to a mere 220 cases, which are expected to represent some of the most reliably detected functionally associated proteins. Using additional information from sequence databases, we have been able to detect pairs of functionally associated proteins with important functions in cellular and developmental pathways, such as spermatogenesis and programmed cell death. Copyright © 2003 John Wiley & Sons, Ltd.**

Since the publication of the first entire genome sequence, hundreds of other genomes have or are in the process of being sequenced (Bernal *et al.*, 2001; Nelson *et al.*, 2000). To adequately process this genomic information and further our understanding of genome structure, function and evolution, computational methods for genome-wide analysis have been developed (Eisenberg *et al.*, 2000). These context-based methods allow the prediction of protein function in entire genomes. One such method is the detection of gene fusion events. Individual proteins found to be both homologous to different regions of a composite, multi-domain protein are predicted to be functionally associated, a result that has been confirmed by experimental information (Enright *et al.*, 1999; Enright and Ouzounis, 2001b; Marcotte *et al.*, 1999a, 1999b; Yanai *et al.*, 2001).

We have chosen to delineate functional associations in the entire genome of *Drosophila melanogaster* using fusion analysis in this extensively studied model organism because of its importance in our understanding of eukaryotic biology. In addition, *Drosophila* can be a key organism for the validation and testing of post-genomic computational methods, because its genome is large, complex (Adams *et al.*, 2000), well-annotated and highly curated (Gelbart *et al.*, 1997) and supported by a vast amount of knowledge accumulated from genetics, molecular biology and, more recently, by large-scale experimentation, such as saturated mutagenesis and expression profiling. The above facts also imply that many of the predictions derived by computational genome analysis should also be testable *in vivo*. Proteins found to be fused in other organisms can be predicted to be functionally associated, where the nature of association can range from direct physical interaction to indirect functional association (Enright and Ouzounis, 2001b). The well-developed genetic analysis of

Copyright © 2003 John Wiley & Sons, Ltd.

*Drosophila* makes it an excellent model organism with which the nature of the predicted interactions can thus be analysed.

We have analysed the *Drosophila melanogaster* genome (13 710 protein sequences) as a query against a database of 23 entire genome sequences (Enright and Ouzounis, 2001b), to identify fused, composite protein homologues (Enright *et al*., 1999). We have found 1981 component genes involved in fusion events, with a total number of 18 654 unique pairwise combinations (31 487 in total) matching composite proteins in any other genome (Figure 1). The full results of this analysis are available at: **http://www.ebi.ac.uk/research/cgg/allfuse/**. These high numbers result from the extensive paralogy of the component proteins, as has been previously noted (Enright *et al*., 1999). In order to focus on the most reliable and specific predictions for detailed evaluation, we have further reduced this number by considering pairs whose components are dissimilar (Smith–Waterman $Z$-score $< 5$) (Enright *et al*., 1999; Enright and Ouzounis, 2001b) and exhibit a low degree of paralogy (number of paralogues $< 4$; Enright and Ouzounis, 2001b). These criteria result in 220 cases that were further analysed manually, aided by imported curated annotations from FlyBase (Gelbart *et al*., 1997) and automatically derived annotations from GeneQuiz (Iliopoulos *et al*., 2000). The 220 selected cases are available at: **http://www.ebi.ac.uk/research/cgg/allfuse/go.html**.

We have previously demonstrated that the method can reliably identify pairs of proteins that potentially interact or are functionally associated (Enright *et al*., 1999; Enright and Ouzounis, 2001b). In the *Drosophila* case, apart from the scale of the analysis, other challenges were erroneous gene definitions (at the time of the analysis) that result in consecutive component gene pairs homologous to a full-length homologue, and the large amount of paralogy which reduces the confidence of the predictions (Enright *et al*.,

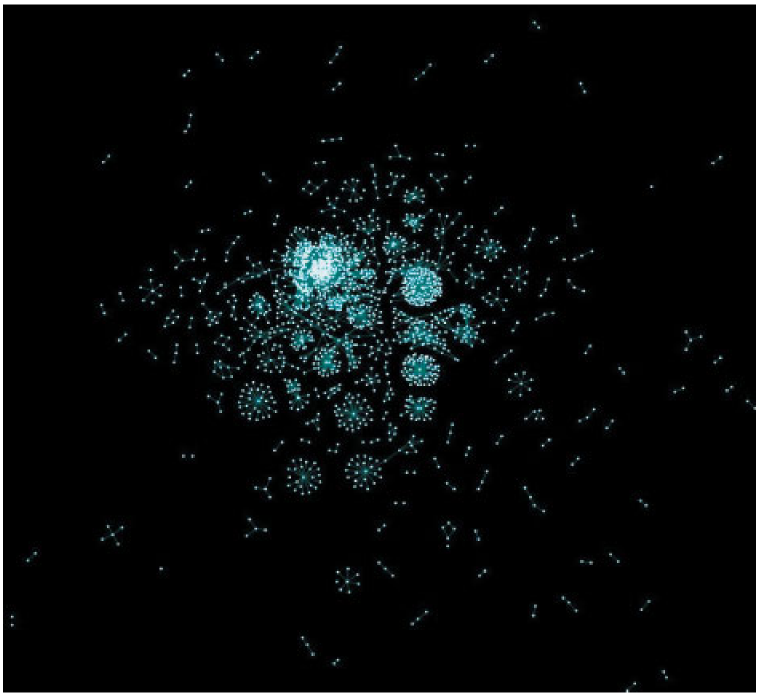

**Figure 1.** A functional association map for the entire genome of *Drosophila melanogaster*. Circles represent 1981 component proteins (nodes) and 18 654 lines (edges) represent functional associations of a component pair on the basis of sequence similarity with another composite protein (Enright *et al*., 1999). All 18 654 unique component pairs are shown. The selection procedure for non-similar components with low degree of paralogy (see text) results in 220 unique component pairs selected out of the entire set. This figure was generated using the 'biolayout' algorithm for automatic graph layout (Enright and Ouzounis, 2001a)

Copyright © 2003 John Wiley & Sons, Ltd.

1999). With more refined gene definitions, it is expected that some of the observed cases (especially component proteins predicted to be encoded by consecutive genes) will be eliminated, thus improving the reliability of our observations (Ashburner, personal communication). In addition, the majority of the cases contain proteins that share homology with the composite protein that is restricted to a short domain only. These cases can be of great interest because the two conserved domains might associate with each other (Enright and Ouzounis, 2001b; Marcotte *et al*., 1999b).

The full, searchable, collection of 18 654 unique pairwise combinations and the 220 filtered cases are accessible over the World Wide Web. Herein, we discuss some of the most interesting cases out of the 220 non-paralogous fusion events; we refer to those with a number available at the corresponding website. Most of the cases presented here involve fusion events whose components have predicted or known functions and represent interesting candidates for experimental verification.

First, there are cases where the identified component proteins (or their homologues) are of known function and known to interact. These cases serve as an internal control of our method. For example, the 49 kDa and 30 kDa subunits of the complex I mitochondrial NADH : ubiquinone oxidoreductase, chain D (EC 1.6.5.3; Belogrudov and Hatefi, 1994) are found to be similar to two composite proteins from *Escherichia coli* and *Aquifex aeolicus* (case 30). Other such examples are the $\alpha$- and $\beta$-subunits of the E1 component of the pyruvate dehydrogenase complex (cases 145 and 147). Finally, a non-trivial case is the detection of methionyl-tRNA synthetase and the aminoacyl–tRNA synthetase auxiliary protein (cases 155 and 161). It has been shown that the latter protein, also called endothelial monocyte activating protein II (EMAP-II) in human, when cleaved from the multisynthetase complex, acts as a cytokine involved in apoptosis (Renault *et al*., 2001; Wakasugi and Schimmel, 1999).

Second, we have identified component proteins whose functions have been characterized in different organisms, but that are not known to interact. Their characterized functions, however, may enable the interpretation of the predicted functional association. One example involves the enzyme trehalose-6-phosphate phosphatase (EC 3.1.3.12) responsible for the dephosphorylation of trehalose-6-phosphate to trehalose and orthophosphate (Strom and Kaasen, 1993) and the enzyme $\alpha$,$\alpha$-trehalase (EC 3.2.1.28), which catalyses the hydrolysis of the disaccharide trehalose (Nwaka and Holzer, 1998) (cases 106 and 107). The homology that these two proteins show to composite proteins from *Mycobacterium tuberculosis* suggests that the two enzymes are directly associated during trehalose metabolism. Another, more intriguing case is represented by the *boule* gene product detected in association with three steroid dehydrogenases (cases 8, 95 and 96), on the basis of their similarity to the *C. elegans* composite protein F56D1.5 (Figure 2). The *boule* gene product has an essential role in spermatogenesis during meiosis, being homologous to the human Y chromosome-encoded azoospermia factor DAZ (deleted in Azoospermia) — both proteins are expressed in the testis (Eberhart *et al*., 1996). The steroid dehydrogenases are highly similar to the human testicular 17$\beta$-hydroxysteroid dehydrogenase (39% identity), an enzyme of testosterone biosynthesis, shown to be responsible for male pseudohermaphroditism (Geissler *et al*., 1994).

Third, the analysis also reveals component pairs where only one of the two genes has been functionally characterized, while the other one is of unknown function. Almost none of these cases have any functional association suspected or observed so far; thus, fusion analysis is a powerful tool that may associate genes of unknown function with characterized genes (Enright *et al*., 1999). One such example is the fly homologue of DAP3, an interferon-$\gamma$-induced positive mediator of cell death in human (Kissil *et al*., 1995), later identified as a mitochondrial ribosomal protein in mouse (Berger *et al*., 2000) (case 60). The other component gene (FlyBase Accession No. *CG12261*) was of unknown function (as of June 2000, at the time when GeneQuiz annotations were generated). By implication to the similarity of these two genes to a composite protein from *C. elegans*, one would deduce that *CG12261* might also be a mitochondrial ribosomal protein, a fact later confirmed by large-scale electrospray tandem mass spectrometry experiments to identify the mammalian mitochondrial small subunit ribosome proteins (Koc *et al*., 2000).

Finally, in the selected list of 220 cases, we have observed many other interesting fusion pairs, involving, for instance, SNARE protein

Copyright © 2003 John Wiley & Sons, Ltd.

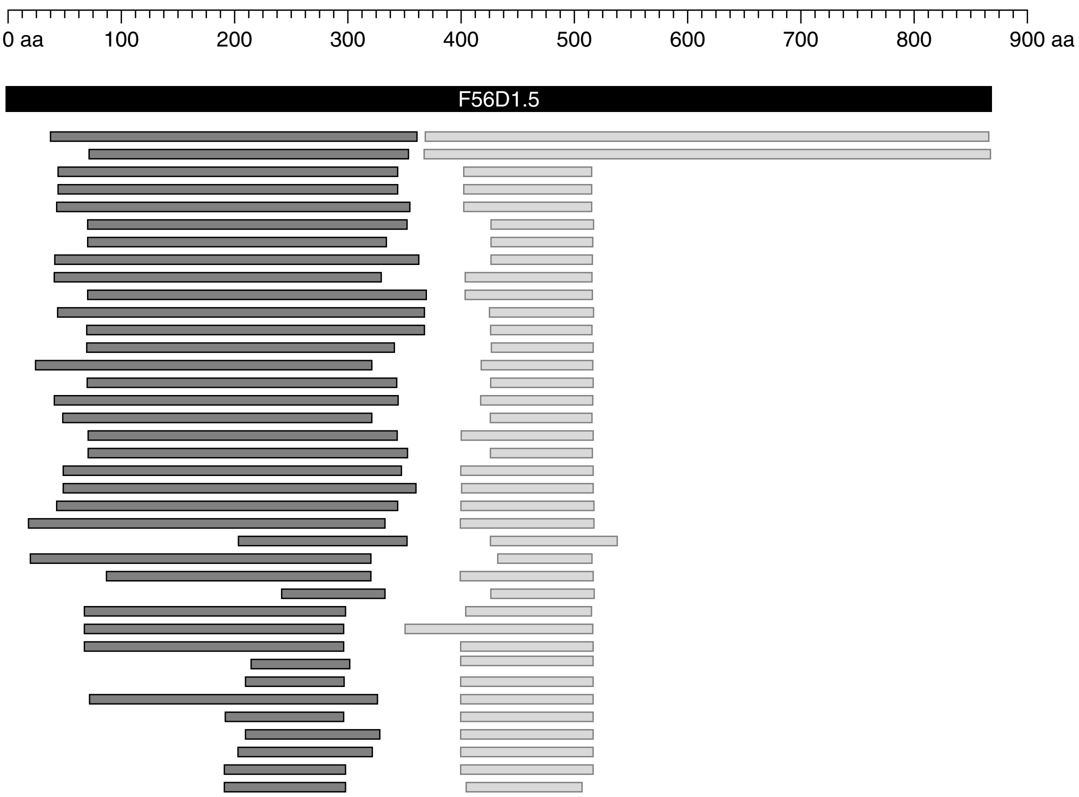

**Figure 2.** Schematic representation of a database search using the *C. elegans* protein F56D1.5 as query (black bar; a scale for amino acid residues is also given). Hits matching the N-terminus of the query protein correspond to a large family of steroid dehydrogenases (represented by dark grey bars); hits matching the C-terminus of the query protein correspond to *boule* homologues (represented by pale grey bars). The pairs do not necessarily belong to the same species. See text for details homologues (cases 10 and 56), Niemann-Pick C disease protein (*NPC1*) (case 45), torsin related protein 4 (*torp4a*) associated with an IMP4 homologue (U3 small nucleolar ribonucleoprotein in yeast) (cases 47 and 48), and the shuttle craft gene product (*stc*) that has been identified as an RS domain protein (Boucher *et al.*, 2001) associated with a NAM7 helicase homologue (cases 63 and 64). Moreover, it is important to emphasize that among the entire list of 18 654 detected fusion pairs, there exist a multitude of other important *Drosophila* genes, such as *Notch, Egfr, trithorax, torso, cactus* and *Delta*. Their detected partners should be interesting targets for further studies using genetics, molecular biology or expression microarray approaches.

To conclude, fusion analysis of a large eukaryotic genome from a model species such as *Drosophila* can shed light on functional associations of gene groups and provide clues about the possible function of uncharacterized genes. The resolution of the method is sufficiently high to allow the generation of testable predictions, especially in such a well-studied organism. As mentioned above, fusion analysis may denote functional associations for a wide spectrum of situations, ranging from genetic to direct physical interactions. This analysis represents a first attempt at a computational derivation of functional associations in a large and complex eukaryotic model organism. Coupled with experimental data from expression profiles, these predictions may be further refined by the scientific community.

### Acknowledgements

We thank Michael Ashburner (University of Cambridge), Benjamin Blencowe (University of Toronto) and Richard

Copyright © 2003 John Wiley & Sons, Ltd. 

Coulson (EBI) for comments, and Joel Anne (DKFZ, Heidelberg), Gunter Merdes (ZMBH, Heidelberg) and members of the Computational Genomics Group for discussions. This work was supported by the European Molecular Biology Laboratory.

### References

Adams MD, Celniker SE, Holt RA, *et al.* 2000. The genome sequence of *Drosophila melanogaster*. *Science* **287**: 2185–2195.

Belogrudov G, Hatefi Y. 1994. Catalytic sector of complex I (NADH : ubiquinone oxidoreductase): subunit stoichiometry and substrate-induced conformation changes. *Biochemistry* **33**: 4571–4576.

Berger T, Brigl M, Herrmann JM, *et al.* 2000. The apoptosis mediator mDAP-3 is a novel member of a conserved family of mitochondrial proteins. *J Cell Sci* **113**: 3603–3612.

Bernal A, Ear U, Kyrpides N. 2001. Genomes OnLine Database (GOLD): a monitor of genome projects world-wide. *Nucleic Acids Res* **29**: 126–127.

Boucher L, Ouzounis CA, Enright AJ, Blencowe BJ. 2001. A genome-wide survey of RS domain proteins. *RNA* **7**: 1693–1701.

Eberhart CG, Maines JZ, Wasserman SA. 1996. Meiotic cell cycle requirement for a fly homologue of human deleted in Azoospermia. *Nature* **381**: 783–785.

Eisenberg D, Marcotte EM, Xenarios I, Yeates TO. 2000. Protein function in the post-genomic era. *Nature* **405**: 823–826.

Enright AJ, Iliopoulos I, Kyrpides NC, Ouzounis CA. 1999. Protein interaction maps for complete genomes based on gene fusion events. *Nature* **402**: 86–90.

Enright AJ, Ouzounis CA. 2001a. BioLayout — an automatic graph layout algorithm for similarity visualization. *Bioinformatics* **17**: 853–854.

Enright AJ, Ouzounis CA. 2001b. Functional associations of proteins in entire genomes by means of exhaustive detection of gene fusions. *Genome Biol* **2**: r0034.0031–r0034.0037.

Geissler WM, Davis DL, Wu L, *et al.* 1994. Male pseudohermaphroditism caused by mutations of testicular 17$\beta$-hydroxysteroid dehydrogenase 3. *Nature Genet* **7**: 34–39.

Gelbart WM, Crosby M, Matthews B, *et al.* 1997. FlyBase: a *Drosophila* database. The FlyBase consortium. *Nucleic Acids Res* **25**: 63–66.

Iliopoulos I, Tsoka S, Andrade MA, *et al.* 2000. Genome sequences and great expectations. *Genome Biol* **2**: i0001.0001–i0001.0003.

Kissil JL, Deiss LP, Bayewitch M, *et al.* 1995. Isolation of DAP3, a novel mediator of interferon-$\gamma$-induced cell death. *J Biol Chem* **270**: 27 932–27 936.

Koc EC, Burkhart W, Blackburn K, *et al.* 2000. A proteomics approach to the identification of mammalian mitochondrial small subunit ribosomal proteins. *J Biol Chem* **275**: 32 585–32 591.

Marcotte EM, Pellegrini M, Ng HL, *et al.* 1999a. Detecting protein function and protein–protein interactions from genome sequences. *Science* **285**: 751–753.

Marcotte EM, Pellegrini M, Thompson MJ, Yeates TO, Eisenberg D. 1999b. A combined algorithm for genome-wide prediction of protein function. *Nature* **402**: 83–86.

Nelson KE, Paulsen IT, Heidelberg JF, Fraser CM. 2000. Status of genome projects for nonpathogenic bacteria and archaea. *Nature Biotechnol* **18**: 1049–1054.

Nwaka S, Holzer H. 1998. Molecular biology of trehalose and the trehalases in the yeast *Saccharomyces cerevisiae*. *Prog Nucleic Acid Res Mol Biol* **58**: 197–237.

Renault L, Kerjan P, Pasqualato S, *et al.* 2001. Structure of the EMAPII domain of human aminoacyl–tRNA synthetase complex reveals evolutionary dimer mimicry. *EMBO J* **20**: 570–578.

Strom AR, Kaasen I. 1993. Trehalose metabolism in *Escherichia coli*: stress protection and stress regulation of gene expression. *Mol Microbiol* **8**: 205–210.

Wakasugi K, Schimmel P. 1999. Two distinct cytokines released from a human aminoacyl–tRNA synthetase. *Science* **284**: 147–151.

Yanai I, Derti A, DeLisi C. 2001. Genes linked by fusion events are generally of the same functional category: a systematic analysis of 30 microbial genomes. *Proc Natl Acad Sci USA* **98**: 7940–7945.

Copyright © 2003 John Wiley & Sons, Ltd. 
